# GnRH agonist long protocol versus GnRH antagonist protocol for various aged patients with diminished ovarian reserve: A retrospective study

**DOI:** 10.1371/journal.pone.0207081

**Published:** 2018-11-07

**Authors:** Ming-Chao Huang, Shu-Ling Tzeng, Chun-I Lee, Hsiu-Hui Chen, Chun-Chia Huang, Tsung-Hsien Lee, Maw-Sheng Lee

**Affiliations:** 1 Institute of Medicine, Chung Shan Medical University, Taichung, Taiwan; 2 Department of Obstetrics and Gynecology, MacKay Memorial Hospital, Hsinchu, Taiwan; 3 MacKay Junior College of Medicine, Nursing, and Management, Taipei, Taiwan; 4 Department of Obstetrics and Gynecology, Chung Shan Medical University, Taichung, Taiwan; 5 Division of Infertility Clinic, Lee Women’s Hospital, Taichung, Taiwan; Zhejiang University School of Medicine First Affiliated Hospital, CHINA

## Abstract

This retrospective analysis compared the efficiency of the gonadotropin- releasing hormone (GnRH) antagonist (GnRH-ant) protocol and the GnRH agonist long (GnRH-a) protocol for patients with diminished ovarian reserve (DOR). A total of 1,233 patients with DOR (anti-Mullerian hormone <1.1 ng/mL) were recruited for this retrospective case-control study. They were divided into two groups according to female age. Younger patients were assigned to POSEIDON group3 (PG3: age ≤35 years); older patients were assigned to POSEIDON group 4 (PG4: age >35 years). All patients with DOR underwent controlled ovarian stimulation and fresh embryo transfer (ET) on day 3. We recruited 283 GnRH-a and 54 GnRH-ant cycles for PG3, and 663 GnRH-a and 233 GnRH-ant cycles for PG4. In PG3, the GnRH-a protocol was associated with a lower ET cancellation rate (30/283 = 10.2% vs. 12/54 = 22.2%, p = 0.018) and a higher live birth rate (7/54 = 13.0% vs. 78/283 = 27.6%, p = 0.024) than the GnRH-ant protocol for the initiated cycles. Furthermore, the GnRH-a protocol was correlated with a higher implantation rate than the GnRH-ant protocol for ET cycles (146/577 = 25.3% vs. 11/103 = 10.7%, P = 0.027). No differences in the ET cancellation rate, live birth rate and implantation rate between GnRH-a and GnRH-ant groups were observed among PG4 patients. In conclusion, the GnRH-a protocol was more effective than the GnRH-ant protocol for young patients with DOR. The low ET cancellation rate and high implantation rate may be related to embryo quality or endometrial receptivity, which warrant further investigation.

## Introduction

Clinical management of patients with poor ovarian responses in assisted reproduction technology (ART) cycles is challenging. The rates of clinical pregnancy and live birth are lower in poor ovarian responders than in age-matched normal responders [1–3]. Selection an ovarian stimulation protocol for such patients is difficult because few oocytes are retrieved in a single controlled ovarian stimulation (COS) cycle [4, 5].

The gonadotropin-releasing hormone (GnRH) antagonist (GnRH-ant) protocol is associated with less gonadotropin and fewer injections than those in the GnRH agonist long (GnRH-a) protocol for normal responders [5–7]. Therefore, the GnRH-ant protocol is considered more cost effective and patient friendly for poor ovarian responders than is the GnRH-a protocol [5, 8]. A recent meta-analysis revealed that the GnRH-ant protocol is correlated with a higher cancellation rate due to poor ovarian response compared with the GnRH-a protocol [9], especially in patients with <4 oocytes in previous COS cycles [10] and in patients with expected poor ovarian response [11], thereby raising the concerns about its effectiveness in poor ovarian responders.

Anti-Müllerian hormone (AMH) and antral follicle count (AFC) are the most reliable ovarian reserve markers for predicting the ovarian response in clinical settings [12–14]. Low AMH levels or low AFC is defined as diminished ovarian reserve (DOR), which suggests that only a few oocytes will be retrieved from these patients in a single stimulation cycle. Consequently, AMH and AFC could indicate a risk of poor ovarian response for individual patient prior to COS. Such patients could benefit from counseling regarding protocol selection [14].

The definition of poor ovarian response by AMH or AFC is inconsistent in the literature. In 2011, to reach a consensus and to benefit future clinical trial and meta-analyses, Bologna criteria were proposed for poor ovarian responders [15]. The Bologna criteria comprise three perspectives: patient age, ovarian reserve markers (AMH or AFC), and the number of retrieved oocytes in previous COS cycles [15]. However, the definition of poor ovarian responders in the Bologna criteria is inapplicable to young patients (<40 years) with DOR in their first ART cycles. To comprehensively analyze the efficiency of various young patients with DOR, a more sensitive classification system is required.

The POSEIDON group further stratified poor or suboptimal ovarian responders to more effectively guide their clinical management [16]. One of the purposes of the POSEIDON criteria was to compare the GnRH analog regimens for poor ovarian responders. Age correlated with embryo euploid rates in ART cycles; consequently, age in the POSEIDON criteria is considered a marker for oocyte quality [16]. POSEIDON group 3 (PG3) and 4 (PG4) comprise patients with low AMH (<1.2 ng/mL) or low AFC (<7) prior to COS who are younger or older than 35 years, respectively. In the present study, we compared the efficiency of the GnRH-ant and GnRH-a protocols for patients with low AMH (<1.2ng/mL) in various age groups according to the POSEIDON and Bologna criteria.

## Material and methods

This retrospective case-control study evaluated the efficiency of the GnRH-ant and GnRH-a protocols for patients with DOR in ART cycles. Medical records related to ART cycles were analyzed from the database of a previous prospective cohort study for the effect of genetic polymorphism in ART cycles. All the data were fully anonymized before we accessed them. Approval from the Institutional Ethics Review Board of Chung Shan Medical University Hospital was obtained for the prospective cohort study (CS-13194 and CS2-14033), which including ~95% of the ART cycles in Lee Women’s Hospital from 2013 to 2015. All the participates signed informed consent for the subsequent analysis of medical records for other studies. We have two physicians, one favors using GnRH-a long protocol for the first or second IVF and the other favors using GnRH-anta protocol for the first or second IVF cycles for most patients. Both physicians are well experienced and achieve similar live birth rates according to the records of our infertility center. The following procedure of oocyte retrieval and in vitro embryo culture is shared by embryologists with fixed rotating schedule in our hospital and thus the following treatment will not influence the comparison of these two protocols.

The recruited patients were divided into three groups according to their age on the day of entry into COS cycles. ART cycles combined with fresh embryo transfer (ET) were included. For patients with diminished ovarian reserve, day 3 ET is our routine practice. If the patients requested blastocyst transfer, we would collect embryos from 2 or more oocyte retrieval. Then we would perform blastocyst transfer in frozen embryo transfer cycles. Such cycles are not included in the present study. Women with uterine factor infertility, severe endometriosis, cycles for gamete donation program, and cycles for preimplantation genetic testing for aneuploidy were excluded.

### GnRH-ant protocols

The GnRH-ant (cetrorelix) protocol was performed as described previously [17]. The decision to start GnRH-ant protocol based upon ultrasound on the stimulation cycle day to assure no dominant (>10mm) follicles nor ovarian cysts appeared in the ovaries. The stimulation process commenced with the administration of 300 IU of human menopausal gonadotropin (hMG, Menopur; Ferring, Saint-Prex, Switzerland) per day for 5 days. Subsequently, the hMG dose was adjusted according to the ovarian response. The GnRH antagonist (0.25 mg of cetrorelix; Cetrotide; Merk Serono, Aubonne, Switzerland) was administered on the sixth day of hMG stimulation. The ovarian response to this therapy was monitored by measuring serial serum estradiol (E2) levels and through transvaginal ultrasonography. We stopped gonadotropin administration after observing two leading follicles (at least 17 mm in diameter) and subsequently administered 250 μg of hCG (Ovidrel; Merk Serono).

### GnRH-a protocol

The women participating in this study followed the GnRH-a protocol as described previously [18]. In brief, the protocol began with daily subcutaneous injections of leuprolide acetate (0.5 mg of LA Lupron; Takeda Pharmaceutics, Germany) during the mid-luteal phase of their prestimulation cycle. Follicular development was stimulated using hMG (Menopur; Ferring) for the first 5 days. Subsequently, the dose of hMG was adjusted according to ovarian response. If two or more follicles reached a maximum diameter of 17 mm, 250 μg of hCG; (Ovidrel; Merk) was administered. Transvaginal oocyte retrieval was performed 34–36 hours after the hCG injection.

### Statistical analysis

Various biological parameters related to ART cycles were compared between the two treatment groups (GnRH-a vs. GnRH-ant) through Student’s t test. The rates of fertilization, ongoing pregnancy and live birth for the three groups were compared using the chi-squared test or Fisher’s exact test, as applicable. All analyses were performed using the Statistical Package for Social Sciences (Version 17.0; SPSS, Chicago, IL). P < .05 was considered to indicate statistical significance.

## Results

We recruited 1,233 women with AMH <1.2 ng/mL; they underwent first or second ART cycles, 946 GnRH-a protocols and 287 GnRH-ant protocols, at Lee Women’s Hospital, Taichung, Taiwan. These cycles were divided into three groups by age (Table 1). The young (≤35 years) patients were assigned to PG3 and patients > 35 years of age to PG4: patients 36–39 years formed PG4A and those >40 years, PG4B. The patients in PG4B satisfied the Bologna criteria.

**Table 1:** Comparison of results of controlled ovarian stimulation (initiated cycles) for patients with AMH <1.2 ng/mL between the GnRH agonist (GnRH-a) and antagonist (GnRH-ant) protocols.

**Women Age**	≤35		36~39		≥40	
**Criteria**	POSEIDON group 3		POSEIDON group 4		POSEIDON group 4, Bologna criteria	
**Group**	PG3		PG4A		PG4B	
**Protocols**	GnRH-a	GnRH-ant	GnRH-a	GnRH-ant	GnRH-a	GnRH-ant
**Initiated ART cycles**	283	54	352	73	311	160
**Age (years)**	33.0±1.9	33.4±1.6	37.6±1.1	37.4±1.2	42.3±2.2	42.6±1.8
**Height (cm)**	159.2±5.0	159.6±5.4	159.5±5.5	159.8±5.7	159.3±5.3	159.3±5.5
**Weight (Kg)**	55.4±8.1	55.6±9.1	56.3±9.0	55.9±10.3	57.2±8.0	56.9±7.3
**BMI (Kg/M**^**2**^**)**	21.9±3.1	21.7±2.7	22.1±3.3	21.9±3.5	22.5±3.1	22.5±2.8
**Basal FSH (IU/L)**	10.2±5.5	9.7±5.5	10.9±6.5	9.6±5.4	11.5±6.4	10.9±6.0
**AMH (ng/mL)**	0.65±0.24	0.58±0.24	0.61±0.26	0.57±0.28	0.57±0.26	0.54±0.26
**E2 on hCG day (pg/mL)**	677±468	711±413	637±506	685±450	533±447	575±452
**P4 on hCG day (ng/mL)**	1.00±2.58	0.89±0.75	1.02±4.65	1.33±3.08	1.23±6.15	1.26±2.85
**LH on hCG day (IU/L)**	2.63±3.07^a^	3.71±3.58^a^	2.79±3.85	3.74±4.89	4.36±7.98^b^	6.61±7.81^b^
**rFSH total dose (IU)**	3298±670^c^	3018±939 ^c^	3533±938	3324±946	4319±1075^d^	3937±1054^d^
**ET cancellation (%)**	30 (10.6) ^e^	12 (22.2) ^e^	67 (19.0)	18 (24.7)	91 (29.3)	41 (25.9)
**No oocyte retrieved (%)**	9(3.2)	3 (5.6)	17 (4.8)	6 (8.2)	29 (9.3)	19(9.9)
**No mature oocytes (%)**	6 (2.1)	4 (7.4)	16 (4.5)	1 (1.4)	17 (5.5)	7(4.3)
**No embryos (%)**	15 (5.3)	5 (9.3)	34 (9.7)	12 (16.4)	45 (14.5)	15(9.9)
**ET rate (%)**	253(89.4)	42 (77.8)	285(81.0)	55 (75.3)	220 (70.7)	119 (74.1)

Data are presented as mean ± SD or number (percentage). For the percentage, the numerator is the number of cycles with ET cancellation, no oocyte retrieved, no mature oocytes, or no embryos; and the denominator is the number of initiated cycles.

^a^, p = 0.021;

^b^, p = 0.003;

^c^, p = 0.009;

^d^, p<0.001 by Student’s t test; and

^e^, p = 0.018 by the chi-squared test.

The ovarian reserve markers (age, AMH levels, and basal FSH levels) were similar between the patients in the GnRH-a and GnRH-ant protocols in each group (Table 1). As expected, the total gonadotropin dose was lower in GnRH-ant treatment group than in the GnRH-a treatment group, especially in PG3 (3018 ± 939 IU vs. 3298 ± 670 IU, p = .009) and PG4B (3937 ± 1054 IU vs. 4319 ± 1075IU, p < .001). All the recruited cycles underwent an ovum pick-up procedure. However, ET cancellation rate was higher in the GnRH-ant protocol than in the GnRH-a protocol in PG3 (12/54 = 22.2% vs. 30/283 = 10.2%, p = .021).

We further analyzed the clinical outcome of ART cycles for patients with DOR after ET (Table 2). The day 3 good embryo rates and embryo numbers per transfer were similar for the GnRH-a and GnRH-ant treatments. PG4B had a significantly lower oocyte maturation rates when the GnRH-ant protocol was applied than when the GnRH-a protocol was applied (76.9 ± 23.4% vs. 82.1 ± 22.4%, p = .045). Only in PG3 did the difference in implantation rates between the GnRH-ant and the GnRH-a protocols reach statistical significance (11/103 = 10.7% vs. 146/577 = 25.3%, p = .027). A borderline high abortion rate with the GnRH-ant protocol in PG3 (4/11 = 36.4% vs. 11/91 = 12.2%, p = 0.054, Fischer’s exact test) was also noted. Nonetheless, the live birth rates (7/42 = 16.7% vs. 78/253 = 30.8%, p = .061) were non- significantly lower for the GnRH-ant treatment than for the GnRH-a treatment in PG3 patients (Table 2).

**Table 2:** Comparison of clinical outcomes between the GnRH agonist (GnRH-a) and antagonist (GnRH-ant) stimulation protocols for embryo transfer (ET) cycles for patients with AMH <1.2 ng/mL.

**Women age**	≤35		36~39		≥40	
**Criteria**	POSEIDON group 3		POSEIDON group 4		POSEIDON group 4, Bologna criteria	
**Group**	PG3		PG4A		PG4B	
**Protocols**	GnRH-a	GnRH-ant	GnRH-a	GnRH-ant	GnRH-a	GnRH-ant
**ET cycles**	253	42	285	55	220	119
**Oocyte number**	4.4±2.5	4.9±2.5	4.0±2.3	4.3±2.2	3.2±1.7	3.5±2.1
**MII number**	3.5±2.1	4.0±2.2	3.2±2.0	3.3±1.8	2.5±1.4	2.5±1.6
**Oocyte maturation rate (%)**	81.6±21.0	81.7±19.5	81.3±20.8	81.0±20.5	82.1±22.4^a^	76.9±23.4^a^
**Fertilization rate (%)**	87.0±18.8	89.9±15.4	87.8±20.4	87.2±22.4	88.5±19.9	90.6±18.4
**Day 3 good embryo rate**	52.4±39.5	54.6±35.9	52.1±40.5	55.7±39.8	43.5±42.0	44.5±42.3
**ET number**	2.3±1.1	2.5±0.9	2.2±1.0	2.2±1.1	1.9±1.0	1.8±1.0
**Implantation rates (%)**	25.3 (146/577) b	10.7 (11/103) b	19.4 (119/613)	16.9 (20/118)	6.6 (27/408)	5.0 (11/218)
**Pregnancy rates per transfer (%)**	36.0 (91/253)	26.2 (11/42)	29.8 (85/285)	25.5 (14/55)	11.4 (25/220)	8.4 (10/119)
**Abortion rates (%)**	12.2 (11/91) c	36.4 (4/11) c	16.5 (14/85)	14.3 (2/14)	60.0 (15/25)	40.0 (4/10)
**Live birth rate per transfer (%)**	30.8 (78/253) d	16.7 (7/42) d	24.9 (71/285)	26.7 (12/55)	4.5 (10/220)	5.0 (6/119)
**Weeks**	37.8±2.2	38.0±2.2	37.9±1.9	37.8±2.1	37.6±1.7	36.5±3.3
**Weight (gm)**	2784±611	2908±370	2863±520	2990±677	2776±331	2890±260

Data are presented as Mean ± SD or percentage (number).

^a^. p = 0.045 by Student’s t test;

^b^. p = 0.027;

^c^. p = 0.032; and

^d^ = 0.061 by Chi-square test.

**Fig 1:**
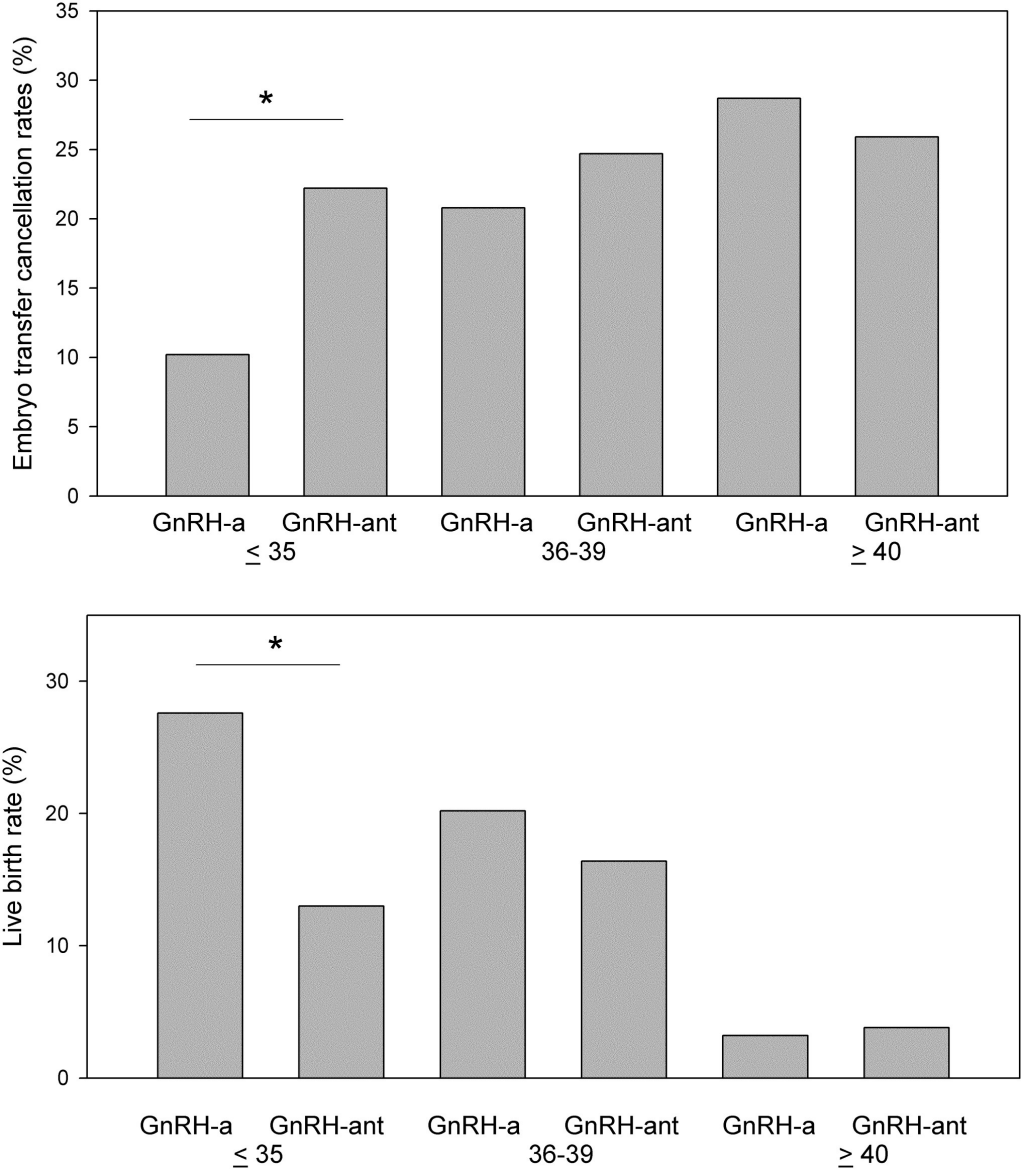
ET cancellation rates and live birth rates per initiated cycle. The numerator is the number of cycles with cancellation or live birth, and the denominator is the number of initiated cycles. * p < 0.05 using chi-squared test.

We explored the outcome of COS and ET for each initiated cycle (Fig 1). In addition to the high cancellation rates in PG3 patients treated using the GnRH-ant protocol (12/54 = 22.2% vs. 30/283 = 10.2%, p = .021), we observed that the live birth rate per initiated cycle was significantly lower in these patients than those treated using the GnRH-a protocols (7/54 = 13.0% vs. 78/283 = 27.6%, p = .024). No difference in ET cancellation rates and live birth rates between the GnRH-ant and GnRH-a protocols were observed in PG4A or PG4B patients (Fig 1).

## Discussion

The present study demonstrated that the GnRH-a protocol was more effective than the GnRH-ant protocol for PG3 patients in terms of lower ET cancellation rates, higher implantation rates, and higher live birth rates. Nonetheless, the efficiency of the two protocols was similar with each other for PG4 patients or poor ovarian responders satisfying the Bologna criteria. The total gonadotropin amount was less when the GnRH-ant protocol was applied than when the GnRH-a protocol was applied for PG3 and PG4B patients.

Clinicians initially recommend the patient-friendly GnRH-ant protocol in COS for patients with poor ovarian response (i.e., reduced AMH levels or low AFC) [5, 8]. One reason is that this reduces the amount of exogenous gonadotropin and the cost of COS [4, 5, 8]. In our study, this benefit was seen in PG3 and PG4B (Table 1). However, if the patients require more COS cycles to achieve a live birth, re-evaluation of the cost-effectiveness of the GnRH-ant protocol for PG3 is necessary.

The reason for a higher ET cancellation rate for the GnRH-ant protocol than that for the GnRH-a protocol for PG3 patients is unclear. A large retrospective analysis for young patients (< 35 years of age) with good prognosis also demonstrated a higher cancellation rate prior to ET for the GnRH-ant protocol than the GnRH-a protocol [19]. In our routine clinical practice and in the most of Taiwan, ovum pick-up is performed even when only one dominant follicle is identified through ultrasound. If the dominant follicles were fewer than three, or slow to develop on day 5, the COS cycle might be canceled in Western countries [10]. A meta-analysis in 2016 indicated a higher cancellation rate due to poor response for the GnRH-ant protocol compared with the GnRH-a protocol [9]; this may, at least partly, explain the high ET cancellation rates for the GnRH-ant protocol in the present study.

Our results indicated low implantation rates for the GnRH-ant protocol for ET cycles in PG3, possibly because of differences in endometrial receptivity. Researchers have suggested that various ovarian stimulation protocols in IVF are linked to endometrial receptivity [20, 21]. An early-stage meta-analysis on pregnancy outcomes revealed that the GnRH-a protocol performed more effectively than the GnRH-ant protocol in 2002 [22]. Patients treated using the GnRH-ant protocol have a thinner endometrium and lower pregnancy rates [23], as well as significantly lower HOXA10 expression in the endometrial stroma cells in patients [24], all of which indicates impaired endometrial receptivity.

By contrast, a study using microarray data revealed that the gene expression profiles of endometrial cells following GnRH-ant treatment are more similar to those in natural cycles compared with those after GnRH-a treatment [25]. Overall, reports on endometrial receptivity for the GnRH-ant or GnRH-a protocols have been inconsistent, although most favor the GnRH-a protocol. If the endometrial receptivity were the principal reason of the difference in implantation rates between GnRH-a and GnRH-ant protocols, the difference should be evident in all three groups because endometrial receptivity is relatively unaffected by age. However, in the present study, only PG3 patients treated using the GnRH-anta protocol exhibited low implantation rates.

Embryo quality or euploid rates are another possible cause of impaired implantation rates in the GnRH-ant protocol. The good embryo rates in the present study did not significantly differ between the two protocols among PG3 patients. Although the oocyte maturation rates were lower in PG4B patients using the GnRH-ant protocol, no difference in the oocyte maturation rates was not observed in PG3. By contrast, a borderline higher abortion rate was observed among PG3 patients treated using the GnRH-ant regimen. The ET cancellation rates for those treated using the GnRH-ant protocol were similar in the three age groups; whereas rates for those treated using the GnRH-a protocol were correlated with age. Zygote score was reported to be associated with pregnancy outcome when the GnRH-ant protocol is applied but not when the GnRH-a protocol was applied [26]. On the basis of these results, we suggest that oocyte (meiosis) maturation and the early stage of embryo development (first mitosis) may differ slightly between oocytes retrieved after the GnRH-ant or GnRH-a protocol was applied. This difference may be related to the embryo implantation potential and may be correlated with age. The embryo euploid rates of these two ovarian stimulation protocols warrant further investigation.

In conclusion, the advantage of the GnRH-a protocol in the present study was evident only among the PG3 patients; the protocol exhibited no advantages for PG4 patients or true Bologna poor ovarian responders. The GnRH-a protocol was associated with a low ET cancellation rate, high implantation rate and high live birth rate. Embryo euploid rate or endometrial receptivity might explain the difference in implantation rate between the GnRH-a and GnRH-ant protocols for young patients with DOR (PG3). Additional large scale randomized control trials are required to confirm the findings of this study.

## Supporting information

S1 Dataset The anonymized data in this study. (XLS)
